# In-Vitro Tooth Cleaning Efficacy and Filament End Rounding of Different Manual Children’s Toothbrushes

**DOI:** 10.3290/j.ohpd.b5573917

**Published:** 2024-07-22

**Authors:** Gina A. Gemperle, Blend Hamza, Raphael Patcas, Marc Schätzle, Florian J. Wegehaupt, Monika A. Hersberger-Zurfluh

**Affiliations:** a Doctoral Student, Clinic of Orthodontics and Paediatric Dentistry, Center of Dental Medicine, University of Zürich, Zürich, Switzerland. Conceptualisation, wrote original draft, performed investigation.; b Head of Division of Paediatric Dentistry, Clinic of Orthodontics and Paediatric Dentistry, Center of Dental Medicine, University of Zürich, Zürich, Switzerland, Conceptualisation, reviewed and edited the manuscript.; c Professor and Scientific Department Head, Clinic of Orthodontics and Pediatric Dentistry, Center of Dental Medicine, University of Zürich, Zürich, Switzerland. Conceptualisation, methodology, reviewed and edited the manuscript.; d Professor, Clinic of Orthodontics and Paediatric Dentistry, Center of Dental Medicine, University of Zürich, Zürich, Switzerland. Conceptualisation, reviewed and edited the manuscript.; e Professor, Head of Department of Preventive Dentistry and Oral Epidemiology, Center of Dental Medicine, University of Zürich, Zürich, Switzerland. Conceptualisation, reviewed and edited the manuscript.; f Senior Teaching and Research Assistant, Clinic of Orthodontics and Paediatric Dentistry, Center of Dental Medicine, University of Zürich, Zürich, Switzerland. Conceptualisation, reviewed and edited the manuscript, methodology, supervised the study.

**Keywords:** children’s toothbrushes, cleaning, efficacy, filament end rounding, paediatric dentistry

## Abstract

**Purpose:**

This in-vitro study aimed to investigate the cleaning efficacy of 18 different manual children’s toothbrushes applying horizontal, vertical, and rotational movements, as well as to evaluate the rounding of their filament ends.

**Materials and Methods:**

Models equipped with artificial teeth (coated with titanium dioxide) were brushed using a brushing machine with clamped manual children’s toothbrushes. The machine carried out horizontal, vertical, and rotational movements for 1 min with a constant contact pressure of 100 g. The percentage of the area of titanium dioxide removed from the buccal, mesial, distal and total surfaces of the artificial teeth corresponded to the cleaning efficacy. To assess the filament design, a scanning electron microscope was used to check the morphology of the filaments, which were scored on the Silverstone and Featherstone scale. SPSS 22 was used for data analysis.

**Results:**

The rotational and the vertical movements achieved the best cleaning efficacy with all tested toothbrushes. The vast majority of the tested toothbrushes had their poorest cleaning efficacy in the horizontal movement. Only a small part of the children’s toothbrushes (3 out of 18) had a correct and acceptable proportion of rounded bristle ends.

**Conclusions:**

Based on the present results, it could be concluded that the cleaning efficacy of different manual children’s toothbrushes varied considerably. The best cleaning efficacy was almost always observed for rotational and vertical movements.

Regular and thorough dental plaque removal is the greatest contributor to the health of the periodontium and dentition. This is typically achieved by adequate toothbrushing with a fluoride-containing toothpaste.^11^^,^^33^ Inadequate and infrequent removal of dental plaque was found to be strongly associated with higher caries prevalence.^15^ Alm^1^ and Elamin et al^10^ found a strong relationship between infrequent tooth brushing at the age of three and high caries experience at the age of 15. Even though a distinct decrease in caries prevalence among children and adolescents was observed over the past decades,^31^ it is still regarded as a common disease within this age group.^1^ For instance, dental caries is the most common chronic disease in children in the United States and it was even reported to be increasing in prevalence in younger children.^7^ The worldwide prevalence of dental caries between 1995 and 2019 was reported to be as high as 46% in primary teeth and 53% in permanent teeth in children.^20^ All these facts attribute to the utmost importance of adequate tooth brushing in children.

Since children usually lack motivation, compliance, and adequate manual dexterity, they are often not able to brush their teeth thoroughly and uniformly.^5^ Here, the support of the parents and caregivers is needed to teach proper brushing techniques and instill thorough tooth brushing habits.^5^^,^^6^ While six different brushing techniques (mostly involving either horizontal, vertical, or rotational movements) are known and recommended by dentists to adults, the horizontal scrubbing method is the most natural technique used by children and it is safe to assume that it is adopted automatically.^5^^,^^25^

Beside the removal of dental plaque, tooth brushing can lead to undesirable side effects. Animal and clinical studies have shown that sharp, unpolished toothbrush bristles might injure gingival tissue.^27^^,^^32^ Other possibly damaging factors are inadequate brushing techniques, the quality and stiffness of the bristles, brushing force, as well as frequency and duration of toothbrushing.^18^ These factors were also connected to damages to tooth hard tissue (i.e., abrasive enamel and dentine wear).^3^^,^^13^^,^^14^ The aim of dental care should be providing optimal plaque removal while preventing injuries of gingiva and tooth hard tissue.

Today, many different designs and modalities of toothbrushes for children are available on the Swiss market. They vary in colour, shape, material, head design, and price. Regardless of these differences, all manufacturers claim their respective toothbrushes to provide optimal plaque removal and cleaning efficacy. For children or their parents, this large number of different varieties can be challenging when deciding on the optimal toothbrush. In this regard, dental professionals should be aware of the different properties of the available toothbrushes and accordingly advise families. The purpose of this study was to investigate the cleaning efficacy of 18 different manual children’s toothbrushes applying common brushing movements (horizontal, vertical, and rotational) under standardised laboratory conditions and a well-established test method,^18^^,^^28^ as well as to assess the rounding of their filament ends.

## MATERIALS AND METHODS 

### Cleaning Efficacy

This study evaluated 18 different types of commercially available manual children’s toothbrushes from ten different manufacturers (Table 1). Photos of the toothbrush heads in addition to their commercially supplied photos were made available on an online repository and can be accessed at (https://doi.org/10.5281/zenodo.8363063). The cleaning performance of these toothbrushes were investigated using a dedicated brushing device (custom-made, ZPZ laboratory, University of Zürich, Switzerland),^18^^,^^28^ which performed three different standardised movements.

**Table:** Table 1 Filament properties of the tested toothbrushes

Toothbrush	Code	Filament length (approx., in mm)	Filament diameter (approx., in mm)	Number of tufts	Number of filaments per tuft	Bristle stiffness according to manufacturer	Brush cut	CE ranking, all surfaces	CE ranking, only proximal
Colgate Kids (6+ y)	COL6	10.50	0.15	27	61 ± 5	soft	contoured	1	4
Elmex Junior (6–12 y)	ELMJ	10.50	0.18	31	53 ± 7	soft	contoured	1	1
Signal Kids with suction cup (0–6 y)	SGN0	9.25	0.15	25	72 ± 4	ultra soft	contoured	3	1
Signal Junior (6+ y)	SGN6	10.75	0.15	31	68 ± 7	ultra soft	contoured	4	9
Candida Lilibiggs Kids (0–6 y)	CAN0	10.0	0.18	22	53 ± 3	soft	plane	5	5
Dentofit Kids (0–6 y)	DFT0	9.50	0.18	26	41 ± 9	sensitiv	contoured	6	3
Colgate Kids (2–6 y)	COL2	9.75	0.15	24	67 ± 7	extra soft	contoured	6	14
Dentamed Kids (1–4 y)	DNTM	8.50	0.17	26	60 ± 11	soft	plane	8	11
Candida Lilibiggs Kids with suction cup mit Saugnapf (3–6 y)	CAN3	10.50	0.13	26	103 ± 7	soft	plane	9	7
Curaprox CS smart (5+ y)	CURS	8.00	0.08	26	292 ± 6	ultra soft	plane	9	11
Dentofit Junior (6+ y)	DFT6	10.25	0.19	37	55 ± 3	sensitive	contoured	11	6
Elmex Kids (3–6 y)	ELMK	10.0	0.15	31	65 ± 5	soft	plane	12	17
Candida Lilibiggs Junior (6–12 y)	CAN6	10.50	0.15	34	68 ± 4	soft	contoured	13	10
Curaprox CuraKid (0–4 y)	CURK	7.00	0.09	26	155 ± 9	supersoft	plane	13	17
Paro S27 (6+ y)	PARS	10.00	0.15	27	62 ± 4	soft	plane	15	14
Trisa Kids (3–6 y)	TRIS	9.75	0.14	27	60 ± 6	soft	contoured	16	8
Oral–B Stages 2 (2–4 y)	ORB2	8.75	0.12	20	99 ± 3	extra soft	contoured	17	11
Oral–B Stages 3 (5–7 y)	ORB5	8.50	0.15	33	61 ± 5	soft	contoured	18	16

y: years. The respective cleaning efficacy (CE) ranking from Tables 2 and 3 was added here to facilitate the comparison between the different properties and the achieved CE. Photos of the tested toothbrushes can be accessed at https://doi.org/10.5281/zenodo.8363063

**Fig 1:**
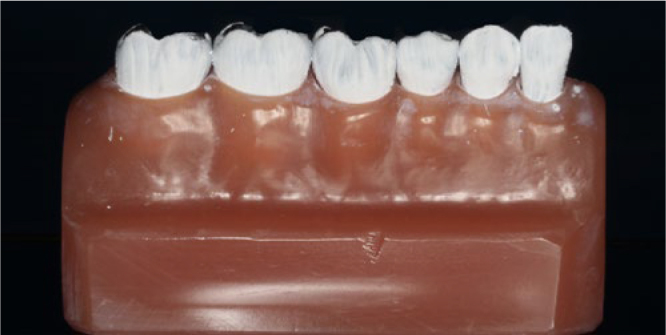
(Fig 1 Experiment model, showing artificial teeth covered with a layer of titanium oxide.

**Fig 2:**
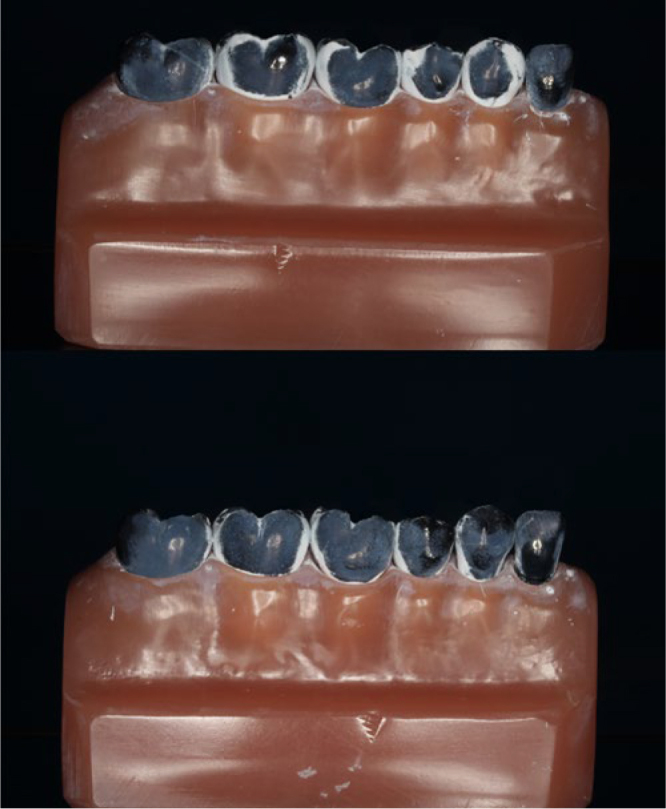
(Fig 2 Tooth surfaces reappearing black after they had been touched by the brush heads and were regarded as potentially cleaned. Top: photo represents a cleaning efficacy of 42%. Bottom: photo represents a cleaning efficacy of 69%.

**Fig 3:**
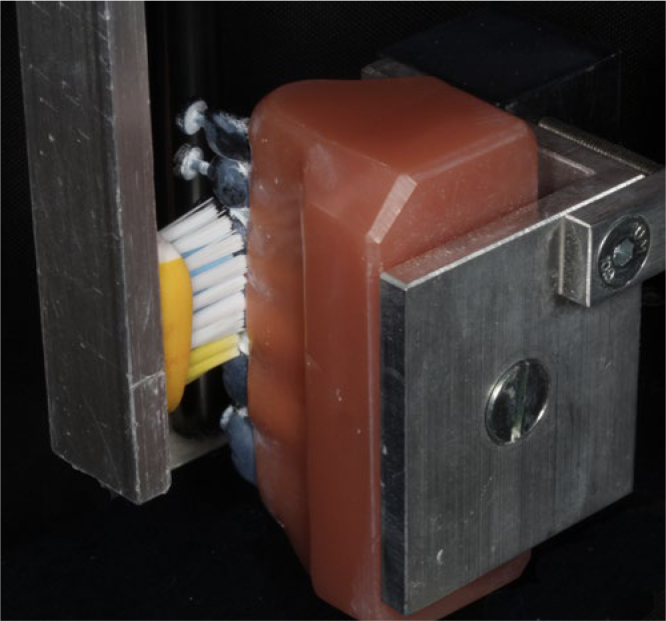
(Fig 3 The moving section of the brushing machine.

To investigate the cleaning efficacy of the toothbrushes, a model of mixed dentition was used. The model consisted of two permanent molars, two primary molars, one primary canine and one primary lateral incisor, all aligned without spaces and all black in colour. The most distal molar and the primary lateral incisor were used for the toothbrush to change direction and were not considered in this analysis. Prior to the experiments, the black model teeth were coated in white using a suspension of titanium oxide in 26vol% ethanol at a ratio of 1:2, simulating 100% plaque accumulation on the tooth surfaces. This powdery coating cannot be peeled off extensively but is removed selectively from sites which are touched by the filaments. Tooth surfaces reappearing black after they had been touched by the toothbrush filaments were regarded as potentially cleaned ((Figs 1 and (2).^18^ The contact pressure for each toothbrush and each movement was 100 g and the brush heads were placed perpendicular to the surfaces of the teeth ((Fig 3).

Horizontal (30-mm excursion/60 strokes), vertical (8-mm excursion/60 strokes combined with horizontal movement of the model 30-mm excursion/16 strokes) and rotational (8x8-mm excursion/60 strokes combined with horizontal movement of the model 30-mm excursion/16 strokes) movements were applied for 1 min each, to simulate the most common brushing techniques. For every movement, a new brush head of the same type was used to prevent the brushes from twisting. Each movement was performed four times on four equal models (4x1 min), and on each model, four teeth were evaluated to reduce bias. Following each round of cleaning, teeth were removed from the model casts and their surfaces were imaged using a scanner (Hewlett-Packard, Palo Alto, CA, USA). For this purpose, teeth were turned while holding them over the platen glass of the scanner, so that curved areas were projected on a plane without distortion. Using specially designed software (custom-made, ZPZ laboratory), gray levels of the scanned teeth were analysed and areas lacking the white coating were recorded quantitatively. The software divided the tooth surface into a buccal, a mesial and a distal area by using a mask and the help of two reference points on each model tooth, which also ensured the superimposition of the mask on the scanned tooth area.^37^ The percentage of cleaned buccal, mesial, distal and total surfaces (black areas) in relation to the entire surface was determined by using the same software.^37^

### Analysis of filament-end rounding

To evaluate the bristle end-rounding quality of the 18 different children’s toothbrushes, five brushes from each type were chosen. From each brush head, five tufts were cut, out of which five bristles were randomly chosen and fixed on a microscope slide. A scanning electron microscope (Amray 1810/T, Amray, Bedford, MA, USA) was used to examine the morphology of the bristles. The bristle ends were evaluated based on the Silverstone and Featherstone scale, as described in the literature, and assigned to one of three different groups which were either correct, acceptable, or inacceptable.^30^ The microscope slides were examined by three different investigators to reduce bias. After the average of all evaluations was calculated and of that a percentage ranking was made. To describe the properties of the toothbrushes, filament length (in mm), filament diameter (in mm), number of tufts, number of filaments per tuft, bristle stiffness and brush cut (planar/flat-trim bristles or contoured/multi-level bristles) were determined for all brushes (Table 1).

### Statistical analysis

Data were analysed using SPSS (IBM SPSS Statistics for Windows, version 24 [IBM; Armonk, NY, USA]). Overall cleaning efficacy, defined as the percentage of cleaned tooth surface area in relation to the entire surface, was descriptively reviewed for all tested brushes, evaluating the three brushing techniques separately. Additionally, approximal cleaning efficacy, defined as the percentage of cleaned mesial and distal tooth surfaces, was similarly descriptively reviewed for all tested brushes, and brushing techniques. All cleaning efficacy scores were ranked according to effectiveness.

Lastly, the quality of the bristle ends of all toothbrushes was analysed and descriptive values were computed (in %) for the respective outcome (correct, acceptable, or inacceptable).

Owing to the large number of different toothbrushes assessed, hypothesis-driven statistics were not performed. Any statistical testing would have yielded p-values that would have had to be adjusted for many multiple comparisons. Testing 18 toothbrushes against each other would result in 153 comparisons, so 153 hypotheses would have to be made and adjusted. This would mainly obfuscate the results without providing any meaningful insights on the cleaning efficacy that are not already discernable based on the descriptive values.

## RESULTS

### Cleaning Efficacy

The cleaning efficacy (%) on all tooth surfaces (buccal, mesial, and distal) for all three tested movements (horizonal, vertical, and rotational) as well as the respective ranking is shown in Table 2. The cleaning efficacy ranged from 31% to 64% for the horizontal movement, 45% to 69% for the vertical movement and 40% to 70% for the rotatational movement. Most of the toothbrushes (14 of 18) exhibited their poorest cleaning efficacy in the horizontal movement. Only ORB2 showed better values in the horizontal movement compared to rotational movement. But none of the tested toothbrushes achieved its best results in the horizontal movement.

**Table 2:** Cleaning efficacy achieved by the tested toothbrushes on all tooth surfaces and their ranking for each tested movement

Toothbrush	Horizontal	Vertical	Rotational	Ranking	Ranking	Ranking	Ranking
	Total surface %	Total surface %	Total surface %	Horizontal	Vertical	Rotational	All movements
COL6	61	69	64	2	1	6	1
ELMJ	64	65	64	1	4	5	1
SGN0	58	63	66	4	6	3	3
SGN6	60	64	61	3	5	11	4
CAN0	57	65	60	6	3	12	5
DFT0	53	63	69	9	8	2	6
COL2	57	63	62	7	7	7	6
DNTM	39	66	66	15	2	4	8
CAN3	51	63	61	10	9	10	9
CURS	58	51	58	5	16	13	9
DFT6	48	55	70	12	15	1	11
ELMK	55	62	55	8	10	15	12
CAN6	43	57	62	14	13	8	13
CURK	50	62	54	11	11	16	13
PARS	34	60	62	17	12	9	15
TRIS	34	57	56	16	14	14	16
ORB2	44	47	40	13	17	18	17
ORB5	31	45	54	18	18	17	18

Ten toothbrushes showed their best cleaning efficacy in the vertical movement and seven did so in the rotational movement. The best performance in the horizontal movement was achieved by ELMJ (64%), in the vertical movement by COL6 with (69%) and in the rotational movement by DFT6 (70%). To compare the overall cleaning efficacy of the tested toothbrushes, an overall ranking was created (Tables 1 and 2), in which the horizontal movement was counted twice as most of children use a horizontal brushing technique.^21^^,^^29^ COL6, ELMJ, SGN0, and SGN6 had the best rankings; they all have a contoured brushing surface.

In addition to the above-mentioned ranking on all tooth surfaces, the cleaning efficacy of the tested toothbrushes only on the approximal surfaces (only mesial and distal) was calculated and ranked for the three brushing movements (horizontal, vertical, and rotational) (Table 3). For the horizontal movement, the cleaning efficacy achieved by the tested toothbrushes ranged from 2% to 16%, 5% to 21% for the vertical movement, and 7% to 35% for the rotational movement. Again, the great majority (16 of 18) of all tested toothbrushes obtained the lowest values in the horizontal movement. The rotational and vertical movements achieved the best cleaning efficacy with all tested toothbrushes (9 of 18 had the best results in the vertical movement, and 7 of them in the rotational movement). For the horizontal movement, ELMJ achieved the best results (16%), for the vertical movement COL6 (21%) and for the rotational movement DFT6 (35%). The ones with the poorest performance were PARS in the horizontal movement (2%), Curaprox CS smart in the vertical movement (5%) and ELMK in the rotational movement (7%). Interestingly, all of them had a flat surface (i.e., all bristles had the same length), while all three brushes with the best results had contoured surfaces.

**Table 3:** Cleaning efficacy achieved by the tested toothbrushes (approximal surfaces only) and their ranking for each tested movement

Toothbrush	Horizontal	Vertical	Rotational	Ranking	Ranking	Ranking	Ranking
	m+d %	m+d %	m+d %	Horizontal	Vertical	Rotational	All movements
ELMJ	16	15	16	1	7	6	1
SGN0	12	16	17	2	6	5	1
DFT0	9	15	24	4	8	2	3
COL6	11	21	14	3	1	10	4
CAN0	5	19	18	12	4	3	5
DFT6	6	8	35	8	16	1	6
CAN3	7	14	13	6	10	12	7
TRIS	4	20	15	14	2	9	8
SGN6	6	15	12	9	9	14	9
CAN6	6	14	15	11	12	8	10
DNTM	2	17	17	17	5	4	11
ORB2	7	13	12	7	13	16	11
CURS	9	5	12	5	18	15	11
PARS	2	20	16	18	3	7	14
COL2	6	10	13	10	15	11	14
ORB5	5	5	13	13	17	13	16
ELMK	3	14	7	16	11	18	17
CURK	3	11	10	15	14	17	17


To compare the overall performance in cleaning the approximal surfaces, another ranking was made using the same formula as described above. ELMJ, SGN0, and DFT0 were ranked as the best.

### Analysis of filament design

The evaluation of the bristle end-rounding quality (%) of all 18 toothbrushes is shown in Table 4. COL2 had the most bristles with correct rounding (68%), followed by ELMJ (57.6%), and DFT6 (55.2%). The two toothbrushes with the most inacceptable (92.8% and 59.2%) and least correctly (1.6% and 10.4%) rounded bristles were Curaprox Curakid and Curaprox CS smart. Noticeably, these were the ones with the thinnest filaments (0.08–0.1 mm) and most filaments per toothbrush head (Table 1).

**Table 4:** Bristle end-rounding quality in % (125 filaments = 100%)

Toothbrush	Correct %	Acceptable %	Inacceptable %
ORB2	48.8	45.6	5.6
CAN0	52.8	39.2	8.0
COL2	68.0	23.2	8.8
ELMK	52.8	36.8	10.4
DFT6	55.2	32.0	12.8
TRIS	54.4	32.8	12.8
PARS	50.4	36.8	12.8
ELMJ	57.6	25.6	16.8
CAN3	44.8	33.6	21.6
DFT0	46.4	29.6	24.0
SGN0	43.2	31.2	25.6
SGN6	44.0	24.8	31.2
CAN6	38.4	30.4	31.2
COL6	40.8	25.6	33.6
DNTM	28.0	30.4	41.6
ORB5	28.8	24.0	47.2
CURS	10.4	30.4	59.2
CURK	1.60	5.60	92.8

## DISCUSSION

Dental caries is the most common dental disease in children across the globe.^24^^,^^26^ Toothbrushing can remove plaque from the surface of the teeth and caries can be prevented. However, the regular use of an unsuitable toothbrush might lead to a poor cleaning performance and fail to the optimally removal of plaque. This study assessed the cleaning efficacy of 18 different manual toothbrushes for children up to age 12 and under applying different brushing techniques, as well as the filament end-rounding quality.

The in-vitro design of the present study represents its main limitation. In fact, an in-vitro brushing setting similar to the one used in the present study has been previously reported and clinically validated by Lang et al.^22^ Those authors compared the clinical cleaning efficacy of two manual toothbrushes to the in-vitro cleaning efficacy of the same toothbrushes (artificial teeth model; teeth covered with artificial plaque staining; inside a brushing machine that conducted horizontal, vertical, and rotational movements). They concluded that the tested robotic toothbrushing can be recommended for the reproducible evaluation of plaque control and cleaning efficacy of different toothbrush designs and brushing actions. Nevertheless, the process of toothbrushing is complicated and cannot be reduced to only brushing movements. Other factors (e.g., the slurry formed by saliva and toothpaste, brushing forces and techniques other than the ones tested here, abrasives and chemical substances within the toothpaste) should also be considered. Therefore, the rankings provided in the present study for the different toothbrushes based on the cleaning efficacy they achieved serve only as a general guide and should not be overinterpreted as the ultimate evaluation of their clinical performance.

Unrounded bristle ends do not necessarily affect the cleaning efficacy of a toothbrush but might cause injuries to the soft tissue.^32^ Additionally, some studies have reported that the risk of soft tissue trauma arises from inappropriate bristle morphology; this risk might increase in handicapped individuals owing to uncontrolled brushing movements.^19^ The same concerns should be applied to healthy children, since individuals under the age of 8 years lack adequate manual skills.^12^ Only three out of 18 toothbrushes (ORB2, CAN0, and COL2) had a correct and acceptable proportion of rounded bristle ends (90% and higher); this proportion in other toothbrushes varied between 7% and 89%.

An earlier study, which investigated the patterns of bristle ends of ten different toothbrushes for children in Korea using a scanning electron microscope and a stereomicroscope, reported the proportion of acceptably rounded bristles to vary between 1.4% to 20.2%.^23^ Another study analysing the morphology of the filament end of eleven toothbrushes for children in Turkey using a stereomicroscope reported the acceptable proportion of filaments to vary between 18.9% and 60.3%.^32^ Differences between the studies are attributed to different tested toothbrushes/manufacturers, whereas different study methodologies might further play a role in the evaluation of acceptable end-rounding. For instance, the percentage of acceptable end-rounding for ORB5 was 52.8% in the present study and 60.3% in the study by Turgut et al.^32^ In the present study, two brushes exhibiting the thinnest filaments (Curaprox CS smart and Curakid: 0.08–0.1mm) had by far the largest proportion of inacceptably rounded bristle endings. However, it cannot be concluded that brushes with thin filaments generally show poor-quality bristle endings, since the brush with the third thinnest filaments (ORB2: 0.12 mm) was able to achieve a proportion of acceptably and correctly rounded bristle endings above 90%. In other words, inacceptably rounded filament ends capable of causing harm do not necessarily correlate with the presence of thin, tapered-end filaments. As a matter of fact, tapered-end filaments could produce more movement flexibility of the toothbrush on gingiva and tooth surfaces. This was confirmed in the study by Versteeg et al,^34^ where a tendency toward less gingival abrasion was observed with the tapered filaments compared to a round-ended ADA reference toothbrush. However, no analysis of acceptably round endings was performed in this study. In a more recent systematic review and meta-analysis, no difference was reported regarding gingival abrasion between tapered filaments and end-rounded ones.^17^ On a different note, Danser et al^8^ observed more gingival abrasion with pointed “gothic-arch shaped” filaments compared to end-rounded ones. In this regard, a tapered-end filament cannot be considered pointed or gothic-arch shaped, as the tapered end of the filament is more attenuated, which provides more flexibility.^13^^,^^34^ To evaluate the effect of different levels of filament end-rounding on gingival abrasion, Hennequin-Hoenderdos et al^16^ tested three manual toothbrushes with different proportions of acceptable filament end-rounding (0%, 40–50% and >90%). The authors concluded that 40%–50% or more acceptably end-rounded filaments provided a statistically significant reduction in gingival abrasion. In this and other studies, gingival abrasion was found to correlate with toothbrushes with sharp filament edges.^2^^,^^4^^,^^16^

Further studies including a higher number of toothbrushes with filaments thinner than 0.1 mm are necessary for clarification. Moreover, no correlation could be established between the brush cut and the quality of the filament end-rounding, as brushes with flat surfaces did not perform better compared to brushes with contoured surfaces.

The horizontal brushing technique is the easiest and by far most intuitive technique for cleaning the teeth, especially for children, because they are still in the process of developing their motor skills. Nevertheless, almost all toothbrushes in the present investigation achieved better results with the vertical and rotational technique compared to the horizontal brushing method, except for the ELMJ, which achieved nearly the same cleaning efficacy with all three techniques. In Switzerland, the vertical technique is taught in schools (IUSP 2020 Interuniversitäre Studiengruppe für zahnmedizinische Prophylaxefragen der Universitäten Basel, Bern, Genf und Zürich); whereas in Germany, the rotational movement is recommended by the Federal Center for Health Education (BZgA). Considering the data of the present study, both recommendations are reasonable, since the cleaning efficacy achieved using these two movements are comparable.

ELMJ, COL6 and SGN0 were always among the top 4 for their cleaning efficacy, regardless of tooth surface (overall or only approximal). All these toothbrushes have a contoured surface (multileveled surface), exhibit between 1600 and 1800 filaments per brush head, with their filaments varying between 9 and 10.50 mm in length and 0.15 to 0.18 mm in thickness. The filament length is important for accessing of the approximal areas, while the variation in filament length (multilevel) is a factor that improves approximal cleaning efficacy.^32^^,^^35^ It can be observed that the toothbrushes with the shortest filaments (e.g., Curakid: 7 mm) performed worse in cleaning approximal areas. Nevertheless, it cannot be concluded that the filament length or length variation is the most decisive factor. Consequently, further investigation is needed to clarify this matter.

No definitive statement about brush cut in correlation with cleaning efficacy can be made based on the results of the present study. However, it was observed that flat brushes with over 2000 filaments per brush (CURS, ELMK and CURK) showed the worst results on approximal surfaces. A possible explanation for the reduced cleaning efficacy of these toothbrushes could be that too many filaments close together mutually impede their flexibility, and the deflection of the filaments is therefore smaller, hence reducing the chance for the filaments to reach the interdental spaces. Similarly, no clear statement about how bristle stiffness correlates to cleaning efficacy can be made based on the present study. All the tested toothbrushes were labelled “soft” (twelve toothbrushes = soft/sensitive; six toothbrushes = super-/ultra-/extra-soft). The toothbrushes with the extra-soft bristles had a scattered distribution in the ranking table (between 3rd and 17th place), suggesting no clear superiority or inferiority of such bristles in comparison to the soft ones. Zimmer et al^36^ reported that manual toothbrushes with hard bristles could achieve better cleaning efficacy but cause more soft tissue trauma compared to toothbrushes with medium and soft bristles. As several toothbrush-related factors (e.g., bristle height, diameter, stiffness, number per tuft and per toothbrush) might affect the resulting cleaning efficacy,^9^^,^^22^^,^^36^ it can be assumed that these factors might mask or alter each other’s effect on cleaning efficacy.

This topic is relevant in that it facilitates parents and their children in choosing the most appropriate toothbrush to prevent soft and hard tissue injuries by sharp filaments. More research is needed to examine the cleaning effects of toothbrushes for children in more detail and identify significant associations. The same applies to the quality of filament end-rounding.

## CONCLUSIONS

Taking the limitations of this study into account, commercially available toothbrushes achieve varying cleaning efficacy, with rotational and vertical movements showing the best results. Most of the tested toothbrushes showed inacceptable end-rounding.
